# Insurance Coverage Policies for Personalized Medicine

**DOI:** 10.3390/jpm2040201

**Published:** 2012-10-30

**Authors:** Andrew Hresko, Susanne B. Haga

**Affiliations:** Duke University, Institute for Genome Sciences & Policy, Durham, NC 27708, USA

**Keywords:** personalized medicine, insurance, adoption

## Abstract

Adoption of personalized medicine in practice has been slow, in part due to the lack of evidence of clinical benefit provided by these technologies. Coverage by insurers is a critical step in achieving widespread adoption of personalized medicine. Insurers consider a variety of factors when formulating medical coverage policies for personalized medicine, including the overall strength of evidence for a test, availability of clinical guidelines and health technology assessments by independent organizations. In this study, we reviewed coverage policies of the largest U.S. insurers for genomic (disease-related) and pharmacogenetic (PGx) tests to determine the extent that these tests were covered and the evidence basis for the coverage decisions. We identified 41 coverage policies for 49 unique testing: 22 tests for disease diagnosis, prognosis and risk and 27 PGx tests. Fifty percent (or less) of the tests reviewed were covered by insurers. Lack of evidence of clinical utility appears to be a major factor in decisions of non-coverage. The inclusion of PGx information in drug package inserts appears to be a common theme of PGx tests that are covered. This analysis highlights the variability of coverage determinations and factors considered, suggesting that the adoption of personal medicine will affected by numerous factors, but will continue to be slowed due to lack of demonstrated clinical benefit.

## 1. Introduction

The field of personalized medicine is anticipated to significantly impact clinical practice as evidence continues to accumulate for new applications that will lead to greater adoption. The excitement surrounding personalized medicine has, in large part, been due to advances in genome-based technologies to assess variation at the DNA, RNA, protein and metabolite level. Already, a number of genomic applications are clinically available that inform medical decision-making and improve health outcomes. In particular, pharmacogenetic (PGx) testing, or tests to predict risk of adverse drug response or likelihood of response, is on the forefront of the personalized medicine movement. 

Despite the potential of personalized medicine and continuing development of new clinical tests, adoption of these tools in practice has been relatively slow. The slow uptake is likely due to a combination of factors including lack of physician knowledge, patient interest, insurance coverage and reimbursement and lack of evidence demonstrating clinical utility [1,2,3,4,5,6]. Regarding insurance coverage, a variety of factors are considered when formulating medical coverage policies, including the overall strength of evidence for a given test, availability of clinical guidelines, current use by physicians, patient interest and cost-effectiveness [7,8]. Underlying each of these factors is the evidence of clinical utility [6,9]. Understandably, payers are reluctant to reimburse a test that may not significantly impact health care decisions and outcomes for a given patient [9]. However, absence of evidence demonstrating clinical utility may be countered by other factors such as physician use [8].

In this study, we evaluated medical coverage policies of the largest U.S. insurers for genomic disease-based tests and PGx tests to determine which tests were covered and the evidence basis for the coverage decisions. This snapshot provides a comprehensive overview of insurance coverage policies for these rapidly developing technologies. Overall, we find that insurers generally agree on their coverage policies for genomic tests, but few tests are currently covered. 

## 2. Methods

To identify coverage determination policies for genomic and PGx tests, we conducted an online search of the top dozen U.S. insurers (based on U.S. News & World Report listing [10]). We excluded two of the companies as they represented local subsidiaries of Blue Cross Blue Shield, instead choosing to focus on companies that insured nationally with single coverage determination policies. The remaining 10 companies included in our search were the Kaiser Foundation Group, Coventry Corporation Group, UnitedHealth Group, Independence Blue Cross Group, Aetna Group, Highmark Group, Humana Group, Wellpoint, HCSC Group, and Cigna Health Group. 

Coverage policies for UnitedHealth Group, Aetna, Humana, Independence Blue Cross Group and Cigna are publicly accessible through the company’s website and search engines. We used the following terms to search for genomic and PGx policies: gene, DNA, mutation and genomic. For the Independence Blue Cross Group, all coverage determinations within the category ‘pathology and laboratory’ for genetic and genomic tests were reviewed (no coverage policies were found in the other categories). We defined genetic and genomic tests to include laboratory analysis of DNA, RNA, or protein. Our search was conducted in August 2012.

If a drug is specifically indicated for patients with a particular genetic aberration, we excluded the tests required for identifying those patients from our analysis. For examples, testing for KRAS (v-ki-ras2 kirsten rat sarcoma viral oncogene homolog for the drugs cetuximab and panitumumab), ALK (anaplastic lymphoma kinase for the drug crizotimib), and Her2/Neu (human epidermal growth factor receptor 2 for the drug trastuzumab) were excluded from our dataset. For these drugs, coverage of testing is likely to be influenced by pharmacy benefits and use of drug without testing would constitute off-label use.

We also reviewed health technology assessments of genomic and PGx tests conducted by two groups: the Centers for Disease Control and Prevention’s Evaluation of Genome Applications in Practice and Prevention (EGAPP) group and the Blue Cross Blue Shield Technology Evaluation Center (BCBS-TEC). For BCBS-TEC, in addition to conducting a text search, we reviewed all assessments in two of the 21 categories listed to identify relevant genomic and PGx assessments: “genetic testing” and “pharmacotherapy/therapy”. For EGAPP, all reports were reviewed. 

Each coverage policy and assessment was reviewed to identify the specific test, evidence considered (if provided) and the policy decision. In addition, we determined whether the test was cleared or approved by the Food and Drug Administration (FDA). For PGx tests, we also determined whether the package insert had been revised to include information about the impact of genetic variants on risk of adverse response or likelihood to respond and PGx testing.

## 3. Results

*Overview of Coverage Policies.* In our search of leading U.S. insurance companies, we identified a total of 41 coverage policies for genomic and PGx testing (summarized in Table 1). Coverage determinations were made for 49 unique tests in the 41 policies analyzed: 22 tests for disease diagnosis, prognosis and risk assessment (Table 2) and 27 PGx tests (Table 3). Although none of the insurance companies covered a PGx test for which the corresponding drug’s package insert had not been updated to include information on the impact of genetic variation, some insurers declined coverage despite revisions to the package insert. Of the 27 PGx tests addressed in the coverage policies, 12 were for drugs whose package inserts had been updated. For disease-related tests, three of 22 tests have been approved by the FDA, of which one is not covered by any of the three insurers that issued a coverage decision on it. In contrast to the PGx tests, many of the disease-related tests analyzed RNA transcripts and typically included analysis of several genes. 

**Table 1:** Summary of Genomic/Pharmacogenetic (PGx) Coverage Policies by Insurer (policies were identified in August 2012 as described in the Methods section).

Insurer	Total # of Policies	Tests for Disease Diagnosis/Risk/Prognosis (% of total review)		PGx Tests	
		# of Tests Reviewed	# of Tests Covered	# of Tests Reviewed	# of Tests Covered
Aetna	8	15	3 (20%)	19	8 (42%)
Independence Blue Cross Group	15	6	3 (50%)	9	2 (22%)
Cigna	8	8	2 (20%)	8	3 (38%)
Humana	7	15	3 (20%)	19	5 (26%)
UnitedHealth	3	5	1 (20%)	0	0 (0%)
TOTAL	41	22 *	4 * (18%)	27 *	8 * (30%)

* Number corresponds to unique tests reviewed or covered.

**Table 2:** Coverage Policies for Disease-related Genomic Tests by Insurer (policies were identified in August 2012 as described in the Methods section).

Test	Disease Indication	Insurer					Tech Assessments		Availability of FDA-approved test?
		Aetna	BCBS	Cigna	Humana	United Health	BCBS TEC	EGAPP	
AlloMap	Cardiac allograft rejection risk	Yes ^1^	Yes ^2^	No ^3^	No ^4^	-	No ^5^		No
CardiaRisk (AGT gene)	Cardiovascular disease risk	No ^6^		No ^7^	No ^4^	-			No
CardioGeneScan test	Diagnostic testing for most cardiac diseases				No ^4^				No
Chromosome 9p21 polymorphism	Cardio vascular disease risk	No ^6^	-	-	-	-	-	Insufficient evidence for or against ^8^	No
Coloprint	Colon cancer recurrence	No ^9^			No ^4^				No
CorusCAD (CardioDx)	Coronary Artery Disease	No ^10^	-	No ^7^	No ^4^	No ^11^	-		No
Decision Dx-GBM	Predictor of progression free survival for glioblastoma				No ^4^				No
Genome Wide Association Screening	Inherited hypertrophic cardiomyopathy	-		-	No ^4^	-			No
HOXB13:ILL7BR Ratio	Breast cancer recurrence risk	No ^9^	-	No ^12^	No ^4^	-	-		No
Interleukin 6–174	Cardiovascular disease risk			No ^7^					No
MammaPrint	Breast cancer recurrence risk	No ^9^	No ^13^	No ^12^	Yes ^4^	-	-	Insufficient evidence for or against ^14^	Yes
Mammostrat	Breast cancer recurrence risk	No ^9^		No ^12^	No ^4^				No
Microsatellite Instability	FAP/Lynch syndrome	Yes ^10^	Yes ^15^	Yes ^16^	Yes ^4^	-	-	Sufficient evidence to recommend testing ^17^	No
My Prognostic Risk Signature™ (MyPRS™)	Predict outcome of newly diagnosed individuals with multiple myeloma	-	-	-	-	No ^11^	-		No
Oncotype DX *	Breast cancer recurrence risk	Yes ^9^	Yes ^13^	Yes ^12^	Yes ^4^	Yes ^11^	No ^18^	Insufficient evidence for or against ^14^	No
Oncotype DX	Colon cancer recurrence risk	No ^9^	No ^13^	No ^16^	No ^4^	No ^11^	-		No
Ovasure/Ovacheck	Ovarian cancer	No ^9^							No
Pathworks Diagnostic Tissue of Origin	Cancers of unknown primary site	No ^9^	-	-	No ^4^		-		Yes
PathfinderTG®	Various neoplasms		No ^19^						No
PreGen-Plus	Colon cancer screening (Stool)	No ^20^							No
Rotterdam Signature 76-Gene Profile	Breast cancer recurrence risk	No ^9^	-	No ^12^	No ^4^	-	-		No
Urovysion	Screening for bladder cancer, hematuria and all other indications	No ^9^							Yes

1. http://www.aetna.com/cpb/medical/data/500_599/0586.html (accessed on 30 August 2012). 2. http://medpolicy.ibx.com/policies/mpi.nsf/e94faffabc7b0da68525695e0068df65/85256aa800623d7a8525786b005929bd!OpenDocument, (accessed on 30 August 2012). 3. http://www.cigna.com/assets/docs/health-care-professionals/coverage_positions/mm_0465_coveragepositioncriteria_blood_tests_heart_transplantation_rejection.pdf, (accessed on 30 August 2012). 4. http://apps.humana.com/tad/Tad_New/Home.aspx, (accessed on 30 August 2012). 5. http://www.bcbs.com/blueresources/tec/vols/26/gene-expression-profiling-as.html, (accessed on 30 August 2012). 6. http://www.aetna.com/cpb/medical/data/300_399/0381.html, (accessed on 30 August 2012). 7. http://www.cigna.com/assets/docs/health-care-professionals/coverage_positions/mm_0137_coveragepositioncriteria_cardiac_disease_risk_laboratory_studies.pdf, (accessed on 30 August 2012). 8. http://www.nature.com/gim/journal/v12/n12/full/gim2010136a.html (accessed on 30 August 2012). 9. http://www.aetna.com/cpb/medical/data/300_399/0352.html (accessed on 30 August 2012). 10. http://www.aetna.com/cpb/medical/data/100_199/0140.html (accessed on 30 August 2012). 11. https://www.unitedhealthcareonline.com/ccmcontent/ProviderII/UHC/en-US/Assets/ProviderStaticFiles/ProviderStaticFilesPdf/Tools%20and%20Resources/Policies% 20and%20Protocols/Medical%20Policies/Medical%20Policies/Gene_Expression_Tests.pdf (accessed on 30 August 2012). 12. http://www.cigna.com/assets/docs/health-care-professionals/coverage_positions/mm_0298_coveragepositioncriteria_assay_genetic_expr_breast_cancer_patients.pdf (accessed on 30 August 2012). 13. http://medpolicy.ibx.com/policies/mpi.nsf/88c0c50066c9d059852574d300564913/d2dd920daca5299e85257839004ea99e!OpenDocument (accessed on 30 August 2012). 14. http://www.egappreviews.org/docs/EGAPPWG-BrCaGEPRec.pdf (accessed on 30 August 2012). 15. http://medpolicy.ibx.com/policies/mpi.nsf/88c0c50066c9d059852574d300564913/85256aa800623d7a852579ba006cb487!OpenDocument (accessed on 30 August 2012). 16. http://www.cigna.com/assets/docs/health-care-professionals/coverage_positions/mm_0014_coveragepositioncriteria_genetic_ testing_for_colorectal_cancer.pdf (accessed on 30 August 2012). 17. http://www.egappreviews.org/recommendations/lynch.htm (accessed on 30 August 2012). 18. http://www.bcbs.com/blueresources/tec/vols/25/gene-expression-profiling.html (accessed on 30 August 2012). 19. http://medpolicy.ibx.com/policies/mpi.nsf/6eeddf656d983ec98525695e0068df68/4554ec7c6fd211ae85257a410063292b!OpenDocument&Highlight=0,pathfinderTG (accessed on 30 August 2012). 20. http://www.aetna.com/cpb/medical/data/500_599/0516.html (accessed on 30 August 2012).

**Table 3:** Coverage Policies for Pharmacogenetic Tests by Insurer (policies were identified in August 2012 as described in the Methods section).

Test	Drug Indication	Insurer					Tech Assessments		FDA Approvals	
		Aetna	Indep-endence BCBS	Cigna	Humana	United Health	BCBS TEC	EGAPP	FDA-cleared test	Revised Drug Label with PGx Info
Apo E	Lipid lowering medications	No ^1^	-	No ^2^	-	-	-	--	No	Yes
BRAF	Cetuximab, pantimumab	-	No ^3^	-	-	-	-	--	Yes	No
Caris TargetNOW Molecular Profiling	Inform cancer therapy	No ^1^	-	-	No ^4^	-	-	--	No	N/A
CYP2C19	Clopidogrel	Yes ^1^	Yes ^5^	No ^6^	No ^4^	-	-	--	Yes	Yes
CYP2C19	Proton Pump Inhibitors	No ^1^	No ^7^	-	No ^4^	-		--	Yes	Yes
CYP2C9/ VKORC1	Warfarin	No ^1^	No ^8^	No ^6^	No ^4^	-	-	--	Yes	Yes
CYP2D6	Tamoxifen	No ^1^	No ^9^	No ^6^	No ^4^	-	No ^10^	--	Yes	Yes
CYP2D6	Tetrabenezine	Yes ^1^	-	-	Yes ^4^	-	-	--	Yes	Yes
CYP2D6	Donepezil	No ^1^	-	-	-	-	-	--	Yes	No
CYP2C9	Proton pump inhibitors	-	-	-	No ^4^	-	-	Insufficient evidence to recommend for or against use ^11^	No	Yes
CYP450 (not specified/multiple)	SSRIs	No ^1^	-	No ^6^	No ^4^	-	-	Insufficient evidence to recommend for or against use ^11^	N/A	N/A
Dihydropyramidine Dehydrogenase (DPYD)	5-Fluorouracil	No ^1^	-	-	No ^4^	-	No ^12^	--	No	Yes
EGFR	Erlotinib	Yes ^1^	No ^13^	-	Yes ^4^	-	Yes ^14^	--	No	No
ERCC1	Cisplatin, carboplatin, oxaloplatin	-	-	-	No ^4^	-	-	--	No	No
HLA-B*1502	Carbamazepine	Yes (in Asian patients) ^1^	-	-	Yes (in Asian patients) ^4^	-	-	--	No	Yes
HLA-B*5701	Abacavir	Yes ^1^	-	Yes ^6^	Yes ^4^	-	-	--	No	Yes
IL28B	Interferon therapy for Hepatitis C	No ^1^	-	-	-	-	-	--	No	Yes (Peg-interferon α2B, Teleprivir, Boceprivir)
KIF6	Statin		No ^15^					--	No	N/A
KRAS	Erlotinib	Yes ^1^	No ^16^	-	No ^4^	-	-	--	Yes	No
MGMT Methylation	Temozolomide (Temodar)	-	-	-	No ^4^	-	-	--	No	No
MTHFR	Antifolate chemotherapy	No ^1^	-	-	-	-	-	Insufficient evidence for or against ^11^	Yes	No
rs3798220	Aspirin	No ^1^	-	-	-	-	-	--	No	No
TPMT	Mercaptopurine, azathiopurine	Yes ^1^	Yes ^17^	Yes ^6^	Yes ^4^	-	-	--	No	Yes
Thymidylate Synthase	5-Fluorouracil	No ^1^	-	-	-	-	No ^18^	--	No	No
Urovysion	Follow-up treatment for bladder cancer	Yes ^1^	-	-	-	--	--	--	Yes	N/A
UTG1A1	Irinotecan	No ^1^	-	No ^19^	No ^4^	-	-	--	Yes	Yes
Whole Genome/Whole Exome/Genome-wide Association study	Pharmacogenetics (not specified)	-	-	-	No ^4^	-	-	--	No	N/A

N/A = not applicable as no individual test or drug specified. 1. http://www.aetna.com/cpb/medical/data/700_799/0715.html (accessed on 30 August 2012). 2. http://www.cigna.com/assets/docs/health-care-professionals/coverage_positions/mm_0137_coveragepositioncriteria_cardiac_disease_risk_laboratory_studies.pdf (accessed on 30 August 2012). 3. http://medpolicy.ibx.com/policies/mpi.nsf/a94c009639ca1eb3852573f4006e01c3/85256aa800623d7a852579a700549ef4!OpenDocument (accessed on 30 August 2012). 4. http://apps.humana.com/tad/Tad_New/Home.aspx (accessed on 30 August 2012). 5. http://medpolicy.ibx.com/policies/mpi.nsf/a94c009639ca1eb3852573f4006e01c3/85256aa800623d7a852579a700549355!OpenDocument (accessed on 30 August 2012). 6. http://www.cigna.com/assets/docs/health-care-professionals/coverage_positions/mm_0500_coveragepositioncriteria_pharmacogenetic_testing.pdf (accessed on 30 August 2012). 7. http://medpolicy.ibx.com/policies/mpi.nsf/a94c009639ca1eb3852573f4006e01c3/85256aa800623d7a852579a7005498a6!OpenDocument (accessed on 30 August 2012). 8. http://medpolicy.ibx.com/policies/mpi.nsf/a94c009639ca1eb3852573f4006e01c3/85256aa800623d7a852579a7005495f8!OpenDocument (accessed on 30 August 2012). 9. http://medpolicy.ibx.com/policies/mpi.nsf/88c0c50066c9d059852574d300564913/85256aa800623d7a852579a700549ac4!OpenDocument (accessed on 30 August 2012). 10. http://www.bcbs.com/blueresources/tec/press/cyp2d6-pharmacogenomics-of.html (accessed on 30 August 2012). 11. http://www.egappreviews.org/docs/EGAPPWG-CYP450Rec.pdf (accessed on 30 August 2012). 12. http://www.bcbs.com/blueresources/tec/vols/24/pharmacogenetic-testing-to.html (accessed on 30 August 2012). 13. http://medpolicy.ibx.com/policies/mpi.nsf/a94c009639ca1eb3852573f4006e01c3/85256aa800623d7a852579a70054a86d!OpenDocument (accessed on 30 August 2012). 14. http://www.bcbs.com/blueresources/tec/vols/25/epidermal-growth-factor.html (accessed on 30 August 2012). 15. http://medpolicy.ibx.com/policies/mpi.nsf/a94c009639ca1eb3852573f4006e01c3/85256aa800623d7a852579a70054a2d2!OpenDocument (accessed on 30 August 2012). 16. http://medpolicy.ibx.com/policies/mpi.nsf/a94c009639ca1eb3852573f4006e01c3/85256aa800623d7a852579a70054a4c5!OpenDocument (accessed on 30 August 2012). 17. http://medpolicy.ibx.com/policies/mpi.nsf/6eeddf656d983ec98525695e0068df68/85256aa800623d7a8525796b007119ee!OpenDocument&Highlight=0,tpmt (accessed on 30 August 2012). 18. http://www.bcbs.com/blueresources/tec/press/pharmacogenetic-testing-to.html (accessed on 30 August 2012). 19. http://www.cigna.com/assets/docs/health-care-professionals/coverage_positions/mm_0381_coveragepositioncriteria_AmpliChip.pdf (accessed on 30 August 2012).

Coverage policies specific to disease-related genomic and PGx tests varied across the insurance companies evaluated. The majority of tests were deemed investigational and not medically necessary. For example, the CYP2C9/ VKORC1 test associated with likelihood to respond and risk of adverse drug response (ADRs) to warfarin was not covered by any of the insurers and deemed investigational. The Oncotype Dx is covered by all insurers to assess breast cancer recurrence risk, but considered investigational with respect to colon cancer recurrence. Twelve of the 49 tests were covered by at least one company, with twice as many PGx tests than disease-related tests covered (Table 1). Nine tests were covered by two or more insurers; one test was reviewed and covered by all insurers (Oncotype Dx). Aetna has reviewed and covers the most tests (closely followed by Humana), and United Health reviewed and covers the fewest tests. Aetna covers the largest number of PGx tests than any other insurer analyzed.

*Basis for Coverage Decisions.* For coverage policies that included background information or reasons for their coverage decision, a review of key clinical studies of the test, clinical guidelines and availability of FDA-approved tests were often included. For several of the tests, while evidence of a strong association between genotype and disease risk or drug effectiveness were noted, the lack of evidence of clinical utility appeared to be the determining factor for coverage. In particular, tests that are not covered tended to lack evidence from prospective, randomized clinical trials. Evidence of cost-effectiveness was not specifically mentioned in any coverage policy. 

*Discordant Coverage Policies.* Of the tests reviewed by more than one insurer, five tests had discordant coverage policies: the Allomap test for cardiac allograft rejection risk, the Mammaprint test for breast cancer risk recurrence, CYP2C19 testing for use of clopidogrel, and EGFR and KRAS testing for erlotinib. The AlloMap test and CYP2C19 PGx test for clopidogrel were reviewed by all insurers except United Health. Aetna and Independence Blue Cross provided coverage for these tests, whereas Cigna and Humana considered both tests to be experimental/investigational and therefore, not covered. 

We compared the coverage policies for discordant coverage decisions. Humana does not provide any background information or rationale for their coverage policies, and therefore, comparisons were not possible. The AlloMap test evaluates expression of 20 genes associated with risk of cardiac allograft rejection following a heart transplant. The test is intended to reduce the number of endomyocardial biopsies performed by identifying patients who are not at risk of rejection, therefore ruling out those patients for biopsy. The BCBS-TEC assessment concluded that the AlloMap test did not meet their criteria for clinical use. Cigna considered a broader range of sources in formulating its policy for AlloMap than other insurers. For example, they commented on four peer-reviewed studies that Aetna did not consider [11,12,13,14]. Two of the four studies considered by Cigna but not Aetna suggested the need for caution in the adoption of the AlloMap test and two provided evidence supporting usefulness of the test. Independence Blue Cross did review and cite those studies, but reached a different conclusion and provided coverage for the AlloMap test. However, both studies showing the test to be effective were limited by uncertain clinical relevance of their findings. Cigna also considered reports of three independent assessments in its review that are not cited by Aetna, including the technology assessment issued by the BCBS-TEC. Aetna considered only one report not cited by Cigna. 

The CYP2C19 test is used to identify patients not likely to respond to treatment with the anti-platelet drug clopidogrel. The prevalence of loss of function mutations in the CYP2C19 gene ranges from 2%–5% in Caucasians and from 13%–23% in people of Asian ancestry [15]. In 2010, the package insert for clopidogrel was revised to include information about the effect of CYP2C19 genetic variants on ADRs and effectiveness. Specifically, a Boxed Warning was added about the reduced effectiveness of clopidogrel in patients who are CYP2C19 poor metabolizers, and to inform health care professionals about the availability of genetic tests for CYP2C19. In addition, a warning was added regarding the concomitant use of a proton pump inhibitors (PPIs) and clopidogrel, since the majority of PPIs are metabolized by CYP2C19 [16].

Comparison of the differing coverage policies between Cigna and Aetna again shows different data were considered. Cigna analyzed nine peer-reviewed articles on genetic testing in use with clopidogrel; Aetna analyzed only one of these studies in its corresponding review but cited (though not discussed) two other studies evaluated by Cigna. Overall, Aetna considered a smaller data set in formulating its policy, analyzing three peer-reviewed articles and citing four others without discussion. One article was cited by both payers without discussion. Five of the six studies evaluated by Cigna but not Aetna suggest an association between CYP2C19 genotype and response to clopidogrel treatment [17,18,19,20,21]. Conversely, both studies evaluated by Aetna but not Cigna question this association, finding no effect of CYP2C19 genotype on response to clopidogrel [22,23]. However, their respective coverage decisions were not supported by the published evidence evaluated. Both insurers considered the American College of Cardiology and the American Heart Association’s guidance about the new “boxed warning” about CYP2C19 poor metabolizers [24]. Aetna also reviewed the 2009 clinical guidelines for percutaneous coronary intervention and management of patients with ST-elevation myocardial infarction issued in conjunction by the American College of Cardiology, the American Heart Association, and the Society for Cardiac Angiography and Interventions. 

## 4. Discussion

One of the important drivers of uptake of genomic tests is insurance coverage. Based on the data collected from a small group of leading insurers, we find that while coverage of genomic and PGx testing is not common, some insurers have been more proactive in this area than others. Overall, 25 percent of the tests reviewed by major insurers are covered, with twice as many PGx tests covered compared to disease-related genomic tests. 

The variability of review and insurance coverage is consistent with other reports [7,8]. The low number of disease-related genomic tests considered for coverage by insurers is likely due to the few studies published demonstrating clinical utility, the often small role of genetics in complex diseases, and availability of alternative effective screening methods. Only one test is reviewed and covered by all insurers, Oncotype Dx, for prediction of breast cancer recurrence. Unlike other tests assessing cancer recurrence risk, prospective clinical studies have been performed demonstrating clinical utility for Oncotype [25]. However, a previous study reported that some insurers offered coverage at an early stage of test development, indicating other factors were considered in their decision that resulted in coverage of the test [8], though not disclosed in the policy. 

While FDA test approval does not appear to be a leading factor in coverage decisions, inclusion of PGx information in drug package inserts does appear to be an important factor. Over the past decade, the number of labels with PGx-related information has increased, including some for commonly used drugs [26,27]. We found that not all tests are covered for drugs with revised package inserts, yet no PGx tests are covered for drugs without revisions to the package insert. Cohen *et al*. [9] similarly reported variability in coverage of PGx tests that are recommended in the package insert. Trosman *et al*. [8] reported that some insurers believed review by the agency indicates that the current evidence was of a sufficient quality for coverage, but also noted that FDA review does not consider evidence of clinical utility that insurers heavily rely upon. For drugs that are indicated for a specific genotype, insurers do not necessarily require proof of testing prior to drug authorization [9]. For disease-related tests, the one test covered by all insurers analyzed, Oncotype Dx, has not been approved by the FDA compared to the limited coverage of a similar FDA-approved test for breast cancer risk recurrence (Mammaprint).

Given that policies are regularly reviewed and updated, we anticipate increased coverage of these tests as evidence is published and physicians become more comfortable ordering such tests, perhaps in response to patient requests, given the high level of interest in PGx testing [28]. As we described earlier, differences were evident in the types of information included in the coverage policies by Aetna. Other factors such as cost-effectiveness and differences in insurers’ coverage pools also likely impact final coverage determinations. In general, payers are optimistic about the use of PGx tests, but advocate for more comparative effectiveness and practical data [29]. As a result of Aetna’s and Humana’s coverage of several PGx tests, this provides an opportunity to collect data regarding utility and cost-effectiveness in a practical clinical setting as it is unlikely that randomized clinical trials will be conducted to provide the evidence that most insurers desire for coverage. In effect, companies offering reimbursement early in the test development process are taking greater risks as well as bearing the cost of evidence generation. 

Another approach to increasing the evidence basis of genomic tests is for the Centers for Medicare and Medicaid Services (CMS) to apply the coverage with evidence development (CED) status to promising genomic and PGx tests [30,31]. This designation encourages use of the test in clinical trials by requiring patient data generation as a condition of coverage [32,33]. CMS has already assigned CED designation to PGx testing for warfarin [34]. CED designation by CMS may encourage other payers to provide reimbursement for promising tests in a similar manor, increasing the rate of evidence generation [35]. The potential of this designation will enable collection of real-time data to assess not only clinical utility in practical settings [3] but assess economic outcomes as well [1].

The early adoption of tests is an important factor considered in coverage decisions, particularly in the absence of published evidence of clinical utility [8]. Therefore, improving physician and patient awareness of tests will be essential for adoption. Little data exists about patient consent of personalized medicine tests, but public interest has been reported to be high [36]. Physician use of select PGx tests appears to be gradually increasing [37,38,39,40,41,42,43]. Despite the increasing prevalence of PGx in drug package inserts, it is unlikely that physicians are aware of these changes and therefore, better notification of revised package inserts may increase consideration of testing for these drugs [44].

In summary, insurance coverage for disease-related genomic and PGx testing is low and variable, though some insurers are willing to provide coverage based on limited evidence of clinical utility. Expanding data collection efforts outside of traditional clinical trial study designs and promoting physician and patient awareness will likely lead to greater review and coverage of these tests. 
